# Exploring the molecular landscape of cancer of unknown primary: A comparative analysis with other metastatic cancers

**DOI:** 10.1002/1878-0261.13664

**Published:** 2024-05-15

**Authors:** Laura Andersen, Ditte S. Christensen, Asbjørn Kjær, Michael Knudsen, Andreas K. Andersen, Maria B. Laursen, Johanne Ahrenfeldt, Britt E. Laursen, Nicolai J. Birkbak

**Affiliations:** ^1^ Department of Molecular Medicine Aarhus University Hospital Denmark; ^2^ Department of Clinical Medicine Aarhus University Denmark; ^3^ Bioinformatics Research Center Aarhus University Denmark; ^4^ Department of Oncology Aarhus University Hospital Denmark

**Keywords:** cancer of unknown primary, genomic profile, immune response to cancer, transcriptomic profile

## Abstract

Cancer of unknown primary (CUP) tumors are biologically very heterogeneous, which complicates stratification of patients for treatment. Consequently, these patients face limited treatment options and a poor prognosis. With this study, we aim to expand on the current knowledge of CUP biology by analyzing two cohorts: a well‐characterized cohort of 44 CUP patients, and 213 metastatic patients with known primary. These cohorts were treated at the same institution and characterized by identical molecular assessments. Through comparative analysis of genomic and transcriptomic data, we found that CUP tumors were characterized by high expression of immune‐related genes and pathways compared to other metastatic tumors. Moreover, CUP tumors uniformly demonstrated high levels of tumor‐infiltrating leukocytes and circulating T cells, indicating a strong immune response. Finally, the genetic landscape of CUP tumors resembled that of other metastatic cancers and demonstrated mutations in established cancer genes. In conclusion, CUP tumors possess a distinct immunophenotype that distinguishes them from other metastatic cancers. These results may suggest an immune response in CUP that facilitates metastatic tumor growth while limiting growth of the primary tumor.

## Graphical abstract

Cancer of unknown primary (CUP) is a metastatic cancer characterized by no detectable primary tumor. By performing transcriptomic and genomic analysis of CUP tumors and metastatic cancers of known primary, this study finds that CUP tumors exhibit elevated expression of immune‐related genes and pathways, higher levels of tumor‐infiltrating leukocytes and circulating T cells, and non‐distinctive mutations in established cancer genes.

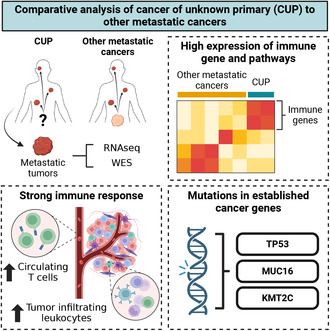

**Abbreviations**
AUH: Aarhus University Hospital
CI: confidence interval
CIN: chromosomal instability
COSMIC: catalog of somatic mutations in cancer
CPI: check‐point inhibitors
CUP: cancer of unknown primary
ESMO: European Society for Medical Oncology
FDR: false discovery rate
GSVA: gene set variation analysis
MSI: microsatellite instability
MSigDB: molecular signature database
OPRA: oncology precision medicine Aarhus
RNAseq: RNA sequencing
SBS: single base substitutions
SCNA: somatic copy number alterations
SD: standard deviation
SNV: single nucleotide variant
TCRA: T cell receptor alpha
TIL: tumor‐infiltrating leukocyte
TMB: tumor mutation burden
TPM: transcript per million
TSG: tumor suppressor gene
WES: whole exome sequencing
wGII: weighted genome integrity index

## 1 Introduction

Cancer of unknown primary (CUP) is a type of metastatic cancer characterized by the absence of a detectable primary tumor, making up 3–5% of all cancer cases worldwide. CUP is diagnosed based on an extensive and unsuccessful search for a primary tumor with standard diagnostic procedures as recently outlined by the European Society for Medical Oncology (ESMO) [1]. Due to the biological and phenotypic heterogeneity of the disease, no consensus treatment currently exists. The standard of care is therefore mainly based on the clinical presentation of the tumor, which leaves the patients with limited treatment options and an often extremely poor prognosis [2]. CUP tumors with pathological profiles similar to known cancer types are treated with standard procedures according to the cancer type of resemblance and these patients have a median overall survival of 10–16 months [3]. This is opposed to patients with no or limited resemblance to known cancer types which are treated as a single entity with platinum‐based chemotherapy. These patients show poor response and have a median overall survival of 6–7 months [2, 3, 4]. As CUP patients often show improved outcomes if treated by organ‐specific protocols, attempts have been made to try to model the likely primary tumor site based on molecular data [5, 6]. However, to this day no definite molecular method has been fully implemented in the clinic, partially due to the difficulty associated with validating the predicted molecular profile [7].

The development of metastatic disease is currently believed to progress in a linear manner with genetic alterations initializing carcinogenesis at a primary site and subsequent proliferation and clonal evolution of the cancer to form a primary tumor. As cancer cells in the primary tumor acquire the ability to metastasize, individual cells or groups of cells disseminate from the primary tumor and colonize to distant sites. These metastatic lesions remain similar to the primary tumor and can be treated with the same type of drugs [8]. With CUP tumors, no primary can be located. It is possible that in the case of CUP, metastasis proceeds as a process of parallel progression with dissemination from the primary tumor at an early stage, followed by clonal evolution of the metastatic site to become distinctly different from the primary site [8]. This hypothesis is one of the current explanations for the origin of CUP where the primary tumor is either too small to be detected, dormant or has spontaneously regressed. Another theory states that CUP is a distinct type of cancer with no primary tumor and molecular characteristics unique to this group of patients [9].

CUP is generally characterized as a cancer with high mutational heterogeneity among tumors, yet frequent genetic alterations in well‐known cancer driver genes, including TP53, EGFR, KRAS, and PIK3CA, are often documented [4, 10]. While most CUP patients are attempted treated with organ‐specific therapy or receives a platinum‐based cocktail, biomarkers associated with positive response to treatment with immune check‐point inhibitors, such as overexpression of PD‐L1, high mutational burden, and microsatellite instability (MSI) have been reported in CUP tumors [11]. However, while some studies have demonstrated promising results [12, 13], immunotherapy is generally not used in CUP patients.

Despite current attempts to understand the biology of CUP, these patients continue to face limited treatment options and poor prognosis. Most studies focus on the classification of CUP into known cancer types in order to stratify them for treatment or characterize the molecular profile of the patient in relation to a particular cancer type. With this work, we compare a cohort of CUP patients treated at Aarhus University Hospital (AUH), Denmark, to a mixed cohort of metastatic cancer patients with known primary site treated at the same unit and subjected to identical molecular procedures. We expand on the current understanding of CUP and demonstrate that CUP tumors share a distinct signature rich on immunological markers. Specifically, we show that the CUP phenotype is characterized by high tumor infiltration of CD8 T cells, but also that CUP patients appear to generally harbor a higher level of T cells in circulation relative to patients with other types of metastatic cancers.

## 2 Materials and methods

### 2.1 Cohort description

A total of 44 patients with CUP treated at Aarhus University Hospital (AUH), Denmark, were prospectively included in this study. Patients were collected from July 2016 to May 2022. RNA sequencing (RNAseq) data was available for 43 patients, and whole exome sequencing (WES) was available for 44 patients. All patients diagnosed with CUP in this study were treatment‐naive, and comprehensive diagnostic procedures were undertaken to identify a primary tumor. The diagnostic protocol commenced with an initial CT‐scan, followed by either colonoscopy or gastroscopy in pursuit of locating a primary tumor. Subsequently, patients underwent a clinically guided search for a primary tumor. The diagnosis of CUP was established when no primary tumor could be identified through the aforementioned diagnostic efforts. Biopsy sites for CUP patients are listed in Table S1.

As a comparative reference, 213 metastatic cancer patients with known primary origin were included. These were collected through the Oncology Precision Medicine Aarhus (OPRA) study at AUH designed as a precision medicine trial for patients out of treatment options. RNAseq data was available for all 213 patients, and WES data was available for 207 patients.

**Fig. 1:**
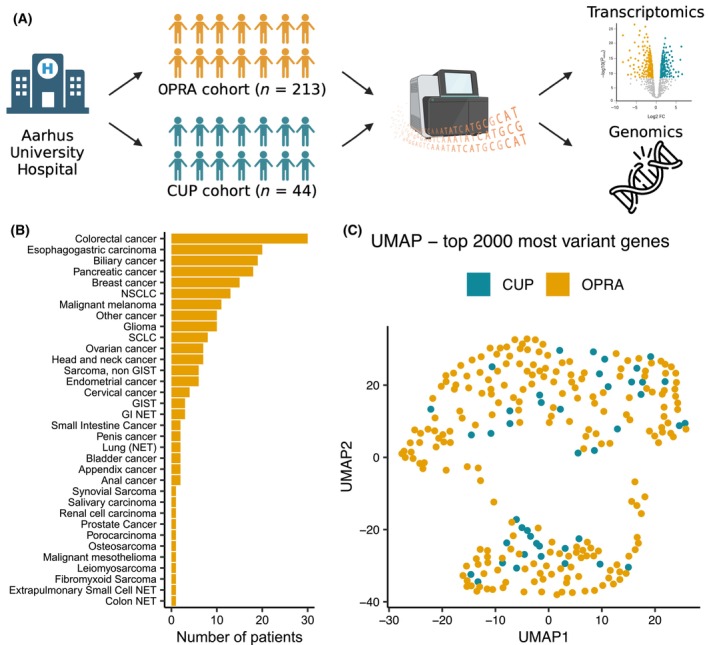
Overview of the cancer of unknown primary (CUP) and Oncology Precision Medicine Aarhus (OPRA) cohorts and workflow. (A) Schematic overview of the two cohorts and the analytical workflow. Created with BioRender.com. (B) Overview of primary cancer types in the OPRA cohort. GI, gastrointestinal; GIST, gastrointestinal stromal tumor; NET, neuroendocrine tumor; NSCLC, non‐small‐cell lung cancer; SCLC, small‐cell lung cancer. (C) UMAP based on the top 2000 genes with highest standard deviation in gene expression across the combined cohorts (CUP: *n* = 43, OPRA: *n* = 213).

The CUP and OPRA cohorts had identical data collection‐ and handling procedures (Fig. 1A). The vast majority of patients in the OPRA cohort had a new biopsy taken and sequenced, primarily from metastatic lesions. In the few cases where a biopsy was either ineligible or the biopsy consisted of normal tissue, the original tissue from the diagnostic biopsy was sequenced and analyzed. Biopsies were core‐needle biopsies or surgical resection samples collected from the same lesion and stored in RNAlater. In addition, blood samples (4 × 10 mL EDTA) were collected for germline mutation analysis. Patients included in the OPRA cohort had metastatic disease with one of 34 different primary cancer types (Fig. 1B) and had been treated according to national guidelines for the given cancer type before inclusion.

Clinical data was available for 44 CUP patients, comprising 25 females and 19 males, with a median age of 67 (confidence interval (CI): 57–70). Additionally, clinical data for 104 OPRA patients was available, including 54 females and 50 males, with a median age of 60 (CI: 50–68).

### 2.2 Ethics approval and consent to participate

The study has been approved by the Committees on Biomedical Research in the Central Region of Denmark (1‐10‐72‐337‐16). All patients provided written informed consent, and the study was performed in accordance with the declaration of Helsinki.

### 2.3 RNA sequencing

Messenger RNA libraries were constructed using ScriptSeq (until nov. 2018, Illumina, San Diego, CA, USA) or KAPA mRNA HyperPrep Kit (Roche) following the manufacturer's directions, applying either KAPA Dual‐Indexed Adapter Kit (Roche, Basel, Switzerland) or xGEN dual index UMI adaptors (Integrated DNA Technologies (IDT), Coralville, Iowa, USA). Paired‐end sequencing (2 × 151) was performed on NextSeq 500 or NovaSeq 6000 (Illumina). In the preprocessing step, sequencing adapters were trimmed using cutadapt [14] (version 4.0) and the trimmed reads were processed to generate count and transcript per million (TPM) expression values with kallisto [15] (version 0.48.0).

### 2.4 Whole exome sequencing

Sequencing libraries from tumor and matched buffy coat DNA were prepared using KAPA Hyper Prep 96/24 Library kit (Kem‐En‐Tek, Taastrup, Denmark) and either KAPA Dual‐Indexed Adapter Kit (Roche) or xGen Dual Index UMI adaptors (IDT) with a minimum input of 500 ng DNA. Libraries were captured using the MedExomePlusV1_hg19 panel (Roche, ~ 71.6 MB). Paired‐end sequencing (2 × 151) was performed on NextSeq 500 or NovaSeq 6000 (Illumina). Sequencing adapters were trimmed using cutadapt [14] (version 3.0) and the trimmed reads were aligned to the reference genome (hg38) using bwa‐mem [16] (version 0.7.17). PCR duplicates were marked and removed with picard [17] (version 2.23.3) MarkDuplicates. Finally, base recalibration and indel realignment was conducted using gatk4 [18] (version 4.1.9.0) IndelRealigner and BaseRecalibrator. Somatic SNVs and INDELs were called using gatk4 [18] (version 4.1.9.0) MuTect2 according to gatk best practices. Variants in coding regions that did not pass the built‐in filters of Mutect2 were retained if they passed the filters of strelka2 [19] (version 2.9.10). ascat [20] version 2.4.2 was used to obtain allele‐specific copy number, purity, and ploidy estimates based on WES data.

### 2.5 Annotation of driver events

All somatic variants were annotated to genes with annovar [21] using hg38 as the reference genome. Driver mutations were called within genes previously associated with cancer, as listed in the cancer gene census defined by the Catalog Of Somatic Mutations In Cancer [22] (COSMIC) database. Individual variants were denoted as driver mutations based on their impact on the protein. For tumor suppressor genes (TSG), driver mutations were defined as frameshift causing indels, nonsense single nucleotide variants (SNVs), splice site SNVs or deleterious SNVs defined as SNVs called as damaging by either sift [23] (‘deleterious’) or polyphen [24] (‘probably damaging’). Additionally, specific mutations that occurred 10 times or more in the driver mutation category defined by the COSMIC [22] database were included. For oncogenes, driver mutations were defined as non‐frameshift indels and SNVs with 3 or more occurrences in the COSMIC [22] v90 database. Finally, for somatic copy number alterations (SCNA) deletions in TSG and amplifications in oncogenes were defined as driver mutations.

### 2.6 Gene expression profile

Transcript per million (TPM) expression values were filtered to include protein coding genes. TPM values were subsequently log2 transformed prior to all analysis. The top 2000 most variant genes based on standard deviation (SD) were used to analyze differentially expressed genes as mean TPM difference between the CUP cohort and the OPRA cohort. Genes with a false discovery rate (FDR) adjusted *q*‐value < 0.25 were used as input to the Reactome Pathway Knowledgebase [25] to search for enriched pathways in both cohorts. The analysis was performed with the ReactomePA R package [26]. Gene set variation analysis (GSVA) was conducted based on all protein coding genes, and a GSVA score was obtained for 50 Hallmark gene sets [27] from the Molecular Signature Database (MSigDB) [28, 29]. These were collected from the msigdbr r package [30]. The analysis was conducted using the gsva function of the GSVA r package [31] with min.sz and max.sz set to 10 and 500 respectively. All other settings were set to default. The mean difference in GSVA score was comparatively analyzed between the CUP and OPRA cohort for each gene set. In the pathway analysis, immune related pathways were defined as being part of the process category ‘immune’ in the Hallmark gene set collection [27], or defined as part of the ‘immune system’ in the Reactome pathway database [25]. To evaluate tumor‐infiltrating leukocytes (TILs), a score was calculated to represent each of 15 leukocytes. This score was calculated based on the mean expression levels of genes associated with distinct immune cell types. The set of genes comprised a total of 67 immune‐related genes, as outlined in Danaher et al. [32].

### 2.7 T cell ExTRECT

Tumor and blood T cell levels were estimated based on WES samples using T cell ExTRECT [33]. T cell ExTRECT estimates the fraction of T cells in a WES sample by quantifying the amount of T cell specific DNA excision in the T cell receptor alpha (TCRA) gene. This is a result of V(D)J recombination during T cell development. T cell ExTRECT was run using the capture probes target regions that overlap TCRA genes and otherwise with default settings. Since the capture kit used for WES highly influences the T cell ExTRECT estimate, some patients were excluded where an older capture kit had been used. In total, we included 33 CUP patients and 191 OPRA patients in the T cell ExTRECT analysis.

### 2.8 Mutational profile

The somatic tumor mutation burden (TMB) was defined as the number of exonic single nucleotide variants (SNV) per megabase for each patient, not including synonymous SNVs. The number of cancer driver mutations were normalized for each patient by dividing the total number of driver mutations with TMB.

Mutational signatures were estimated from the mutation profile of each sample based on the trinucleotide context of single base substitutions (SBS) using deconstructsigs (version 1.9.0) [34]. The mutational signatures were filtered to include those where 10 or more patients had a signature fraction larger than 0. This was done to exclude signatures with predominantly zero values from the statistical analysis. The total count of each mutational signature was calculated based on the signature fraction multiplied with TMB for each patient.

Mutations in immune genes were defined as driver mutations in genes defined as part of the ‘immune system’ in the Reactome pathway database [25].

The weighted genome integrity index (wGII) was calculated based on the available segmented copy number data, as previously described [35].

### 2.9 Statistical analysis

All analysis was performed using r version 4.1.2. Statistical analysis was performed using the ggpubr r package [36]. Wilcoxon ranked sum test was used to test for differences between groups. False Discovery Rate (FDR) were used to adjust *P*‐values for multiple testing. Fisher's exact test was used to test for association of mutations between the two cohorts. Uniform Manifold Approximation and Projection (UMAP) was performed with the umap r package [37]. Heatmaps were constructed using the ComplexHeatmap r package [38]. Throughout the analysis, a *P*‐value below 0.05 is considered significant unless stated otherwise.

## 3 Results

### 3.1 CUP tumors do not form a transcriptionally distinct group

To characterize the molecular landscape of CUP, we assembled a cohort of 44 patients diagnosed with CUP and 213 patients with metastatic disease of known primary tumor (Fig. 1A). These were enrolled in a precision medical trial at AUH, OPRA, and provided both transcriptomic and genomic data (Section 2). The OPRA cohort consisted of patients with 34 different primary cancer types (Fig. 1B). Both cohorts were sampled and processed at the same hospital, making them ideal for comparative analyses. To investigate whether CUP tumors were distinct from OPRA tumors in their overall transcriptomic profiles, we performed UMAP analysis based on the top 2000 most variant genes (Fig. 1C). We observed that CUP tumors showed no distinct separation from metastatic tumors of known origin, indicating that CUP shares molecular similarities with other metastatic cancers rather than representing a distinct cancer subtype. We further repeated the analysis using all protein coding genes and assessed the primary cancer types of the OPRA cohort (Fig. S1). As previously observed for metastatic cancers [39], the OPRA cohort did not form clusters according to tissue of origin. Additionally, as shown above, the CUP tumors did not form a distinct cluster, suggesting that CUP is not a unique phenotype based on their transcriptomic profile.

### 3.2 Higher expression of immune‐related genes and pathways in CUP patients

**Fig. 2:**
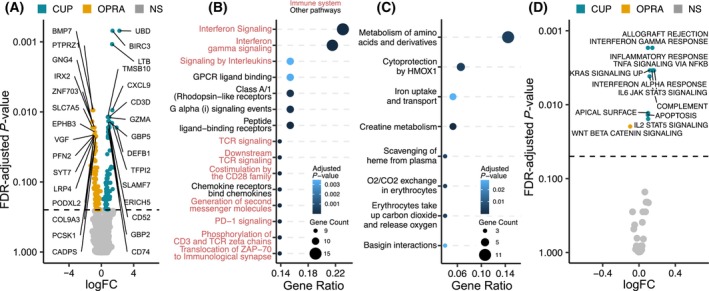
Differential expression analysis between the cancer of unknown primary (CUP) (*n* = 43) and the Oncology Precision Medicine Aarhus (OPRA) cohort (*n* = 213) on gene and pathway level. (A) Differential gene expression analysis performed on the top 2000 most variant genes. The *x*‐axis shows the log2 fold change (logFC) in transcript per million (TPM) values between the CUP and OPRA cohort. The *y*‐axis shows the two‐sided false discovery rate (FDR) adjusted *P*‐value. Genes with an FDR *q*‐value < 0.25 are colored blue and yellow for increased expression in CUP and OPRA respectively. Top 15 genes with highest *P*‐value in each cohort are labeled. Non‐significant (NS) genes are colored gray. Dotted line indicates *P*‐value = 0.25. (B) Reactome pathway enrichment analysis based on the 91 genes with significantly higher expression in the CUP cohort from (A). The *x*‐axis shows the fraction of genes involved in the given pathway. The *y*‐axis shows the significant pathways. Pathway name in red indicates immune related pathways. Dots are colored according to the adjusted *P*‐value. Size indicates the number of genes mapped to a specific pathway. (C) Same as (B) but for the 119 genes with significantly higher expression in the OPRA cohort from (A). Dotted line indicates *P*‐value = 0.05. (D) Differential pathway expression analysis based on the Hallmark gene sets. The *x*‐axis shows the logFC in gene set variation analysis (GSVA) score between the cohorts. The *y*‐axis shows the two‐sided FDR adjusted *P*‐value. Pathways with FDR adjusted *q*‐values < 0.05 are colored blue and yellow for increased expression in CUP and OPRA respectively. NS pathways are colored gray. Wilcoxon ranked sum test was used for comparison in (A) and (D).

To explore potential expression patterns distinguishing CUP tumors from OPRA tumors we first performed differential gene expression analysis on the top 2000 most variant genes. Among the genes that demonstrated significant differential gene expression between the cohorts, 91 genes were upregulated in CUP tumors, while 119 genes were upregulated in OPRA tumors (FDR *q*‐value < 0.25, Fig. 2A, Table S2). Reactome pathway enrichment analysis of these genes revealed that CUP tumors were enriched in pathways related to the immune system (Fig. 2B), whereas no coherent pattern was observed in the pathways enriched in the OPRA cohort (Fig. 2C). To further investigate the differences in expression profiles between the CUP and OPRA cohorts on a pathway level, we performed Gene Set Variation Analysis (GSVA) [31] using the Hallmark [27] gene sets (Fig. 2D, Table S3). Of the 11 pathways significantly enriched in CUP, seven were related to the immune system. One pathway was found to be enriched in OPRA.

**Fig. 3:**
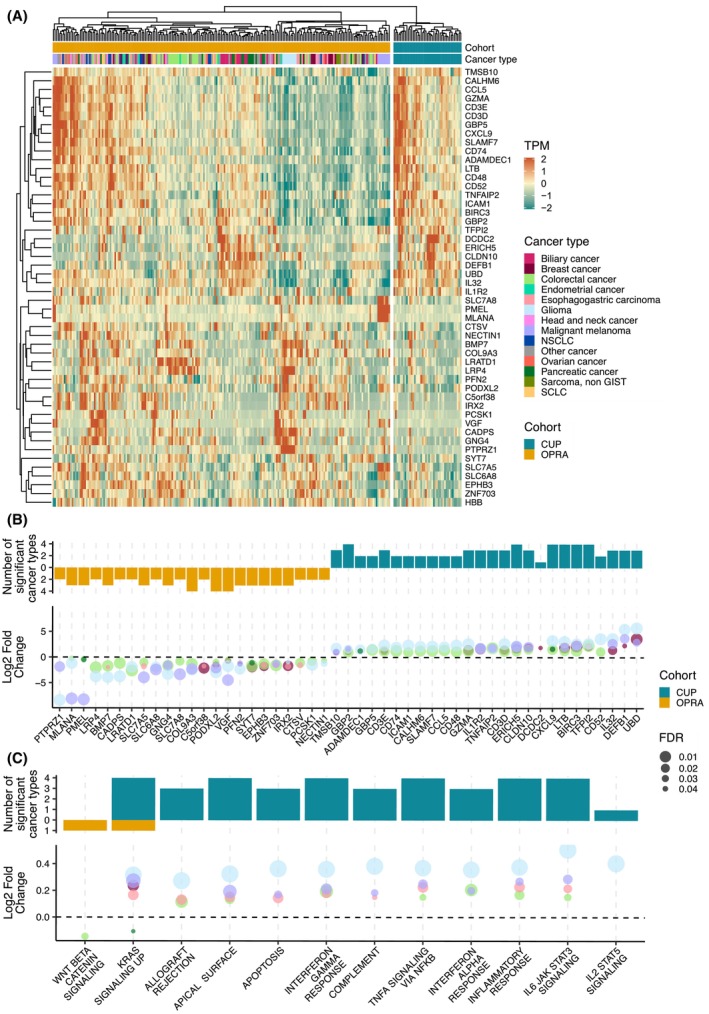
Cancer type specific comparison of differentially expressed genes and pathways. (A) Heatmap of transcript per million (TPM) for the top 50 genes with highest false discovery rate (FDR) *q*‐value in the differential gene expression analysis from Fig. 2A. The heatmap is split by the cancer of unknown primary (CUP) (*n* = 43) and Oncology Precision Medicine Aarhus (OPRA) (*n* = 213) cohorts and each cohort is clustered based on the 50 genes. Cancer type and cohort is annotated at the top. (B) Cancer type specific comparison of the 50 genes from (A) between CUP and distinct cancer types of OPRA with 10 or more patients. These cancer types include colorectal cancer, esophagogastric carcinoma, biliary cancer, pancreatic cancer, breast cancer, non‐small‐cell lung cancer (NSCLC), malignant melanoma and glioma. Top figure shows the number of cancer types where the specific gene was found to be significantly increased in CUP (blue) or increased in OPRA (yellow). Bottom figure shows the TPM log2 fold change (logFC) between CUP and the given cancer type. Size indicates FDR *q*‐value (Wilcoxon ranked sum test) and color indicates primary cancer type (see legend from A). (C) Same as (B) but for Hallmark gene sets with significant differential expression between CUP and OPRA from Fig. 2D.

The observed immune transcriptional profile in CUP tumors may potentially be caused by an over‐represented immune‐cold cancer type within the OPRA cohort. However, clustering analysis of the top 50 most significant genes from Fig. 2A did not reveal any specific cancer type with lower gene expression compared to CUP patients (Fig. 3A). While some OPRA tumors exhibited TPM values distinct from CUP tumors, this pattern did not appear to be driven by a singular cancer type.

To further expand on these findings, we conducted pairwise comparison of gene expression levels between CUP tumors and individual cancer types within the OPRA cohort, including cancer types with 10 or more patients (biliary cancer, breast cancer, colorectal cancer, esophagogastric carcinoma, glioma, malignant melanoma, non‐small‐cell lung cancer and pancreatic cancer). Among the 26/50 genes enriched in CUP compared to all of OPRA, six genes demonstrated significant enrichment compared to four cancer types in OPRA, 10 genes to three cancer types, and nine genes to two cancer types (Fig. 3B). Of the genes enriched in OPRA, 23 were significant in at least two cancer types (Fig. 3B). These observations suggest that the enriched genes observed in CUP are not driven by a single cancer type.

To repeat the analysis on a pathway level we compared GSVA scores of significant pathways from Fig. 2D to individual cancer types in OPRA. We found 6/7 of the enriched pathways in CUP to be significantly different compared to at least three cancer types in OPRA (Fig. 3C). Only colorectal cancer exhibited increased expression in WNT beta catenin signaling compared to CUP.

Taken together, these findings suggest that the immune system may play an important role in the development of CUP metastasis, with CUP tumors demonstrating increased expression of immune related genes and pathways compared to other metastatic cancers.

### 3.3 CUP tumors are immunologically hot

**Fig. 4:**
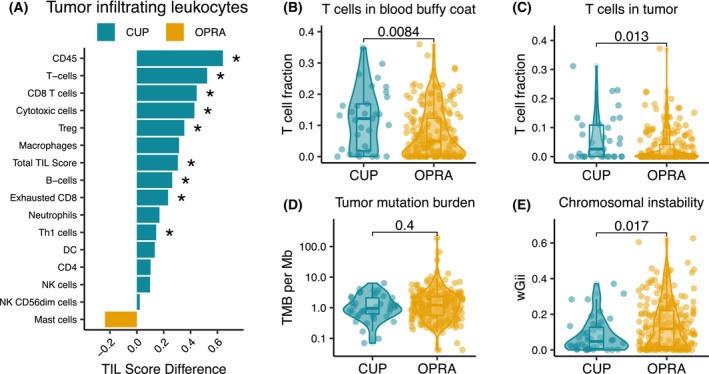
Immunological landscape and summary of mutation status between the cancer of unknown primary (CUP) and Oncology Precision Medicine Aarhus (OPRA) cohort. (A) Comparison of tumor‐infiltrating leukocytes (TILs). The *x*‐axis shows the mean TIL score difference between the CUP (*n* = 43) and OPRA (*n* = 213) cohort. * indicates false discovery rate (FDR) adjusted *q*‐value < 0.05. (B) Comparison of the T cell ExTRECT T cell fraction based on whole exome sequencing (WES) data in blood buffy coat between CUP (*n* = 33) and OPRA (*n* = 191). (C) Same as (B) but in the tumor. (D) Comparison of tumor mutation burden (TMB) between CUP (*n* = 44) and OPRA (*n* = 207). The *y*‐axis shows the TMB per megabase. (E) Comparison of chromosomal instability between CUP (*n* = 44) and OPRA (*n* = 207). The *y*‐axis shows the weighted genome integrity index (wGII). Wilcoxon ranked sum test was used for all comparisons.

To further explore the immunological landscape of CUP tumors relative to OPRA tumors, we performed immune cell deconvolution on the RNAseq data. We utilized the method defined by Danaher et al. [32] to evaluate the relative abundance of 15 immune cell types in the CUP and OPRA cohorts (Section 2). We found that 13 out of 15 immune cell types exhibited higher levels in CUP tumors; eight of which were significantly higher (CD45, T cells, CD8 T cells, Cytotoxic cells, Treg, B‐cells, Exhausted CD8 and Th1 cells) along with the total TIL score (Fig. 4A). Notably, all immune cell types with a significant increase in CUP tumors comprised components of the adaptive immune system, particularly T cell subtypes. Additionally, we found increased levels of exhausted T cells in CUP compared to biliary, colorectal, breast cancer and glioma (Fig. S2), suggesting an elevated presence of exhausted T cells in CUP.

To further investigate the immunological aspects of CUP tumors we implemented T cell ExTRECT, a method recently developed by Bentham et al. [33] to estimate the overall sample T cell content based on WES data. We performed T cell ExTRECT analysis on buffy coat and tumor samples from 33 CUP and 191 OPRA patients (Section 2). We observed that both tumor and buffy coat samples from the CUP cohort had significantly higher levels of T cells than the OPRA cohort (buffy coat: *P* = 0.0084, tumor: *P* = 0.013, Fig. 4B,C). While this was only significant for malignant melanoma (both buffy coat and tumor) and breast, colorectal, and other cancer (tumor), a trend towards decreased T cell levels was observed in all cancer types, except for Biliary cancer (Fig. S3).

Taken together, this shows that CUP tumors exhibit an immune‐hot phenotype, characterized by infiltration of cytotoxic immune cells. However, this immune response appears to be insufficient in clearing the cancer, potentially attributed to an elevated presence of exhausted T cells.

### 3.4 Tumor mutation burden and chromosomal instability

The adaptive immune system identifies cancer cells by recognizing neoantigens presented on their cell surface. In several cancer types, a high number of neoantigens have been associated with a positive response to immunotherapy [40]. As TMB is known to correlate with the number of neoantigens [41], we evaluated TMB in both cohorts. Surprisingly, although CUP tumors showed a significant increase in immune cell infiltration compared to OPRA tumors, we found no significant difference in TMB between the two cohorts (*P* = 0.4, Fig. 4D). This suggests that the difference in immune cell infiltration is not driven by an increased number of neoantigens in CUP tumors.

As chromosomal instability (CIN) has previously been associated with tumor inflammation and aggressive cancer [42], we further investigated whether the overall level of CIN differed between the CUP and OPRA tumors. For this analysis, we used the weighted genome integrity index (wGII) [43] to estimate a summary score of CIN. Interestingly, we found a lower level of CIN in CUP tumors compared to OPRA tumors (*P* = 0.017, Fig. 4E). Taken together, these results indicate that increased immune infiltration in CUP tumors is not driven by higher levels of genomic alterations or increased levels of CIN.

### 3.5 The most prevalent driver mutations in CUP affect well‐established cancer genes

As mutations in specific genes might potentially drive early metastasis, we endeavored to explore the mutational landscapes in the CUP and OPRA cohorts. For this analysis, we called driver mutations across a set of genes previously defined as involved in cancer (Section 2). We did not observe any driver mutations to be significantly more mutated in CUP tumors relative to OPRA tumors (Fig. S4A). In both cohorts, the most mutated gene was TP53, followed by MUC16, KMT2C and CSMD3. These are well‐established cancer genes and among the most commonly mutated genes in cancer in general. We further evaluated the number of driver mutations relative to TMB (Section 2) and found no significant difference between the two cohorts (*P* = 0.16, Fig. S4B).

To address whether the increased immune system in CUP could be explained by mutated immune genes, we evaluated mutations in immune‐related genes. We defined immune‐related genes as being part of the immune system in the Reactome pathway database [25]. No specific immune gene was found to be more mutated in CUP compared to OPRA (Fig. S4C). CUP tumors displayed a slight decrease in the relative number of immune gene mutations compared to OPRA tumors (*P* = 0.043, Fig. S4D).

These findings suggest that the mutational landscape is similar between CUP and OPRA, and that no specific gene appears to drive the metastatic process in CUP. Furthermore, no immune gene mutations were found to explain the inability of the immune system to contain metastatic CUP tumors.

### 3.6 Mutational processes are similar in CUP and OPRA tumors

Different mutation‐generating processes are known to affect the genome in distinct patterns based on trinucleotide context of individual variants. These patterns are known as mutational signatures [44]. To explore whether mutagenic processes differ between the CUP and OPRA cohorts, we conducted a mutational signature analysis (Section 2, Fig. S4E). Surprisingly, our analysis revealed no significantly enriched mutational signatures in either of the cohorts, suggesting that the mutation‐generating processes are similar between the CUP and OPRA cohorts.

Overall, our study provides evidence that CUP tumors have a distinct immunological profile compared to metastatic cancers with known primary origin. While we observe an increased presence of adaptive immune cells, the inability of the immune system to eliminate the cancer cells could potentially be explained by higher levels of exhausted CD8 T cells. These findings suggest that CUP tumors are primarily distinguished from other metastatic cancers by an increased immune infiltration at metastatic sites.

## 4 Discussion

To the best of our knowledge, this study is the first to compare tumor genomic and transcriptomic data from CUP patients to a mixed cohort of patients with metastatic cancer of known origin. The primary focus of previous research on CUP has been to identify the primary cancer type based on mutations and expression profiles. However, since ~ 80% of CUP tumors do not resemble known cancer types and this group of patients continue to face poor prognosis [3], analyzing CUP as a distinct entity might reveal what differentiates them from other metastatic cancers. Furthermore, such analysis might contribute to the accumulating knowledge of the development of metastatic cancers in general.

The main finding of our study is that CUP tumors present with a unique immunophenotype compared to other metastatic cancers. This is supported by three key findings: firstly, CUP tumors showed an increased expression of immune related genes and pathways, while no immune association was found in OPRA tumors. This distinction held true when comparing CUP to individual cancer types within the OPRA cohort. Secondly, CUP tumors show higher levels of T cells in both the tumor and buffy coat. Lastly, 14 out of 15 TILs trended towards higher presence in CUP tumors, of which the significantly increased ones were subtypes of the adaptive immune system. While the adaptive immune system is generally known to recognize cancer cells by binding neoantigens presented on the cell surface, our findings did not suggest that the increased presence of the adaptive immune system could be attributed to increased TMB. Furthermore, we found no mutations in immune‐related genes to explain a potential immune escape mechanism. In contrast, OPRA tumors displayed increased CIN compared to CUP tumors, a phenomenon previously linked to high tumor inflammation. This contradicts previous findings suggesting that CUP tumors are characterized by high CIN compared to other metastatic cancers [45]. However, it may be attributed to the selection of OPRA patients as these are highly progressed cancers. Further studies are needed to resolve this issue.

We note that while the observed increase in T cells in CUP overall may be a response to a more immune‐hot cancer, it may also be a result of decreased immune capacity in OPRA caused by prior chemotherapeutic treatment.

Since the entry of immunotherapy treatment with check‐point inhibitors (CPI), there has been a strong focus on understanding the role of the immune system in cancer development, growth, and metastatic dissemination. The use of CPI in a clinical setting has dramatically improved patient survival for certain cancer types, particularly metastatic melanoma [46, 47]. Given the critical role of cytotoxic T cells to mediate CPI response through active cancer cell killing, they have received special attention and been the focus of intense research. Particularly, there have been trials of novel immunotherapy regimens such as extracting the patient's own cytotoxic T cells, selecting and expanding T cell clones with high cancer cell specificity, and then re‐administering the expanded T cell population [48, 49].

In our work we found that the immune cell types that were significantly more present in CUP tumors were all subtypes of T cells, including exhausted CD8^+^ T cells. These T cells have been exposed to continuous antigen stimulation causing them to enter a dysfunctional and exhausted state. It is plausible that the mechanisms of T cell exhaustion facilitate an immune escape mechanism in CUP tumors which aids the metastatic dissemination early during the development of the disease. This would explain why CUP tumors are able to evade the immune system despite the increased presence of immune cells. Considering these findings, further investigation of CPIs as a therapeutic option for CUP patients to potentially reinforce the immune response may be beneficial for the patients. Biomarkers associated with CPI response, including the expression of genes linked to CPI response, high TMB, and suspected tissue of origin known to be responsive to CPI, have been identified in CUP patients [50]. Moreover, a phase II study administering Nivolumab to CUP patients demonstrated a response rate of 22% [13].

Further exploration of the immune landscape in CUP tumors may identify potential barriers hindering effective CPI response. This may involve a more comprehensive understanding of immunosuppressive mechanisms and identification of biomarkers to predict response or resistance to CPI in the specific context of CUP. Furthermore, exploring strategies to boost the T cell population could be relevant, paving the way for more targeted and effective CPI approaches in this unique and heterogeneous cancer.

Aside from the increased immune cell infiltration in the CUP cohort, we found no other molecular associates with CUP tumors. While we did detect mutations in well‐established cancer genes in the CUP cohort, which have also previously been reported in studies of CUP [4, 51], we did not identify driver mutations or immune gene mutations that were differentially mutated between the two cohorts. Additionally, we found no difference in mutational signatures between the cohorts. This suggests that no unique genomic alterations drive the metastatic process in CUP, similar to recent findings in metastatic cancer of known origin [52, 53].

Finally, we observed that CUP tumors did not form distinct clusters relative to other metastatic cancers on a transcriptomic level, suggesting that CUP is not a distinct cancer phenotype. It has previously been shown by Robinson et al. [39] that metastatic lesions in general do not cluster into tissue‐of‐origin, hence we cannot conclude whether CUP overlaps with specific cancer types. Previous studies have shown that CUP tumors are genetically highly heterogeneous. Möhrmann et al. [51] compared a cohort of CUP patients to the cancer genome atlas (TCGA) dataset and found that CUP tumors did not form a distinct cluster. Instead, they resembled cancer types that did not cluster distinctly, such as gastrointestinal tumors. Likewise, Vikeså et al. [45] classified CUP tumors into tissue‐of‐origin and found that they on a molecular level were more distantly related to their predicted tumor type than the paired metastatic lesions. These findings suggest that the lack of clustering in CUP is attributed to the genetic heterogeneity, hence highlighting the need for more personalized treatment options than what is implemented today.

A limitation of this study is the small size of the CUP cohort resulting in reduced statistical power, which makes it difficult to overcome the clinical heterogeneity observed in the disease. Additionally, the OPRA patients had been treated according to standard of care of their respective cancer type prior to the initial project biopsy, whereas all patients in the CUP cohort were initially treatment naïve. However, from the time of inclusion into either the CUP or OPRA cohort, all data collection and handling procedures were identical between the two cohorts, as were diagnostic tests and biopsy‐guided treatment protocols.

## 5 Conclusions

Conclusively, our results suggest that CUP tumors have a unique immunological profile that distinguishes them from other metastatic cancers. Specifically, we observe upregulation of immune related pathways and increased tumor infiltrating of immune cells. These findings suggest that CUP patients may possess an immune response that allows metastatic dissemination while remaining effective towards a primary tumor that is undetectable by medical imaging technologies. Our work expands on the current understanding of the biology of CUP and contributes with novel findings. Furthermore, it invites additional investigation of personalized treatment options for CUP patients, potentially with immunotherapy.

## Conflict of interest

The authors declare no conflict of interest.

## Author contributions

LA, DSC, BEL and NJB: conceptualization and methodology, LA, DSC and AK: formal analysis and investigation, MK, AKA, and MBL: resources, LA, DSC, AK, MK, AKA, MBL, and JA: data curation, LA and DSC: writing – original draft preparation, LA, DSC, AK, BEL and NJB: writing – review and editing, LA, DSC and AK: visualization, BEL and NJB: funding acquisition and supervision. All authors approved of the submitted manuscript.

### Peer review

The peer review history for this article is available at https://www.webofscience.com/api/gateway/wos/peer‐review/10.1002/1878‐0261.13664.

## Supporting information


**Fig. S1.** UMAP based on gene expression of all protein coding genes in the cancer of unknown primary (CUP) and Oncology Precision Medicine Aarhus (OPRA) cohorts.
**Fig. S2.** Comparison of exhausted CD8 T cells between cancer of unknown primary (CUP) patients and distinct cancer types of Oncology Precision Medicine Aarhus (OPRA) with 10 or more patients.
**Fig. S3.** Comparison of T cell fractions between cancer of unknown primary (CUP) and distinct cancer types in Oncology Precision Medicine Aarhus (OPRA) with 10 or more patients.
**Fig. S4.** Mutational landscape of the cancer of unknown primary (CUP) (*n* = 44) and Oncology Precision Medicine Aarhus (OPRA) (*n* = 207) cohort.
**Table S1.** Overview of biopsy sites for the cancer of unknown primary (CUP) patients (*n* = 27).
**Table S2.** Differentially expressed genes between the cancer of unknown primary (CUP) (*n* = 43) cohort and the Oncology Precision Medicine Aarhus (OPRA) (*n* = 213) cohort.
**Table S3.** Differentially expressed Hallmark gene sets between the cancer of unknown primary (CUP) (*n* = 43) and Oncology Precision Medicine Aarhus (OPRA) (*n* = 213) cohort.

## Data Availability

The raw sequencing data generated in this study are not publicly available as this compromises patient consent and ethics regulations in Denmark. Processed non‐sensitive data are available upon reasonable request from the corresponding author. The analysis code used for carrying out the visualizing analysis is available on request.
