# Supplementary material for: Exploring the molecular landscape of cancer of unknown primary: A comparative analysis with other metastatic cancers
Source: Mol Oncol. 2024 May 15;18(10):2393–406. doi: 10.1002/1878-0261.13664 (PMC11459033; doi:10.1002/1878-0261.13664)
Supplement: Supplementary file 1 — Fig. S1. UMAP based on gene expression of all protein coding genes in the cancer of unknown primary (CUP) and Oncology Precision Medicine Aarhus (OPRA) cohorts. Fig. S2. Comparison of exhausted CD8 T cells between cancer of unknown primary (CUP) patients and distinct cancer types of Oncology Precision Medicine Aarhus (OPRA) with 10 or more patients. Fig. S3. Comparison of T cell fractions between cancer of unknown primary (CUP) and distinct cancer types in Oncology Precision Medicine Aarhus (OPRA) with 10 or more patients. Fig. S4. Mutational landscape of the cancer of unknown primary (CUP) (n = 44) and Oncology Precision Medicine Aarhus (OPRA) (n = 207) cohort. Table S1. Overview of biopsy sites for the cancer of unknown primary (CUP) patients (n = 27). Table S2. Differentially expressed genes between the cancer of unknown primary (CUP) (n = 43) cohort and the Oncology Precision Medicine Aarhus (OPRA) (n = 213) cohort. Table S3. Differentially expressed Hallmark gene sets between the cancer of unknown primary (CUP) (n = 43) and Oncology Precision Medicine Aarhus (OPRA) (n = 213) cohort. [file MOL2-18-2393-s001.zip › mol213664-sup-0001-FigureS1.pdf]

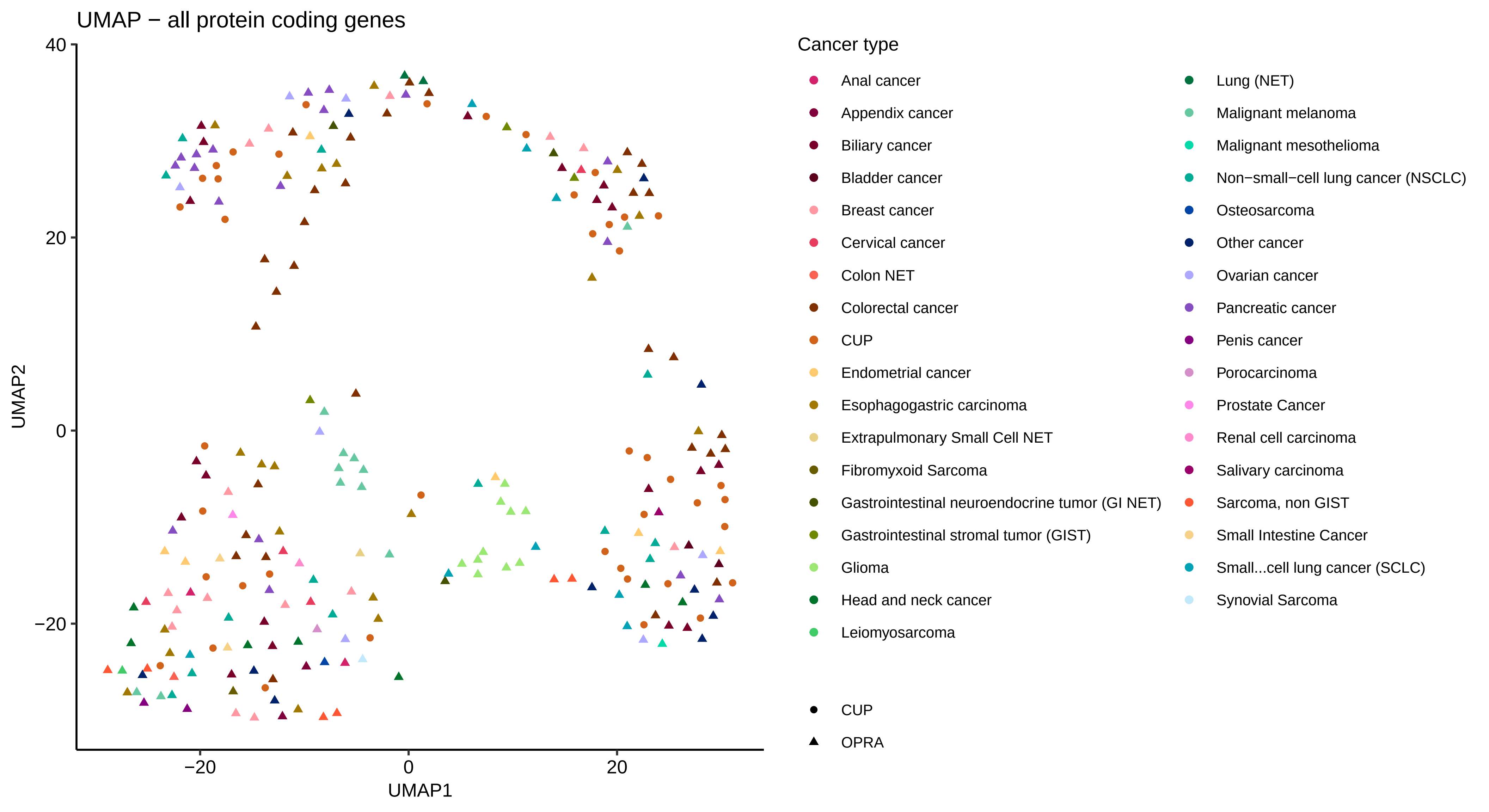
UMAP − all protein coding genes
40
20
0
−20
UMAP2
−20
0
20
UMAP1
Cancer type
Anal cancer
Appendix cancer
Biliary cancer
Bladder cancer
Breast cancer
Cervical cancer
Colon NET
Colorectal cancer
CUP
Endometrial cancer
Esophagogastric carcinoma
Extrapulmonary Small Cell NET
Fibromyxoid Sarcoma
Gastrointestinal neuroendocrine tumor (GI NET)
Gastrointestinal stromal tumor (GIST)
Glioma
Head and neck cancer
Leiomyosarcoma
Lung (NET)
Malignant melanoma
Malignant mesothelioma
Non−small−cell lung cancer (NSCLC)
Osteosarcoma
Other cancer
Ovarian cancer
Pancreatic cancer
Penis cancer
Porocarcinoma
Prostate Cancer
Renal cell carcinoma
Salivary carcinoma
Sarcoma, non GIST
Small Intestine Cancer
Small...cell lung cancer (SCLC)
Synovial Sarcoma
CUP
OPRA
