# Supplementary material for: Exploring the molecular landscape of cancer of unknown primary: A comparative analysis with other metastatic cancers
Source: Mol Oncol. 2024 May 15;18(10):2393–406. doi: 10.1002/1878-0261.13664 (PMC11459033; doi:10.1002/1878-0261.13664)
Supplement: Supplementary file 1 — Fig. S1. UMAP based on gene expression of all protein coding genes in the cancer of unknown primary (CUP) and Oncology Precision Medicine Aarhus (OPRA) cohorts. Fig. S2. Comparison of exhausted CD8 T cells between cancer of unknown primary (CUP) patients and distinct cancer types of Oncology Precision Medicine Aarhus (OPRA) with 10 or more patients. Fig. S3. Comparison of T cell fractions between cancer of unknown primary (CUP) and distinct cancer types in Oncology Precision Medicine Aarhus (OPRA) with 10 or more patients. Fig. S4. Mutational landscape of the cancer of unknown primary (CUP) (n = 44) and Oncology Precision Medicine Aarhus (OPRA) (n = 207) cohort. Table S1. Overview of biopsy sites for the cancer of unknown primary (CUP) patients (n = 27). Table S2. Differentially expressed genes between the cancer of unknown primary (CUP) (n = 43) cohort and the Oncology Precision Medicine Aarhus (OPRA) (n = 213) cohort. Table S3. Differentially expressed Hallmark gene sets between the cancer of unknown primary (CUP) (n = 43) and Oncology Precision Medicine Aarhus (OPRA) (n = 213) cohort. [file MOL2-18-2393-s001.zip › mol213664-sup-0004-FigureS4.pdf]

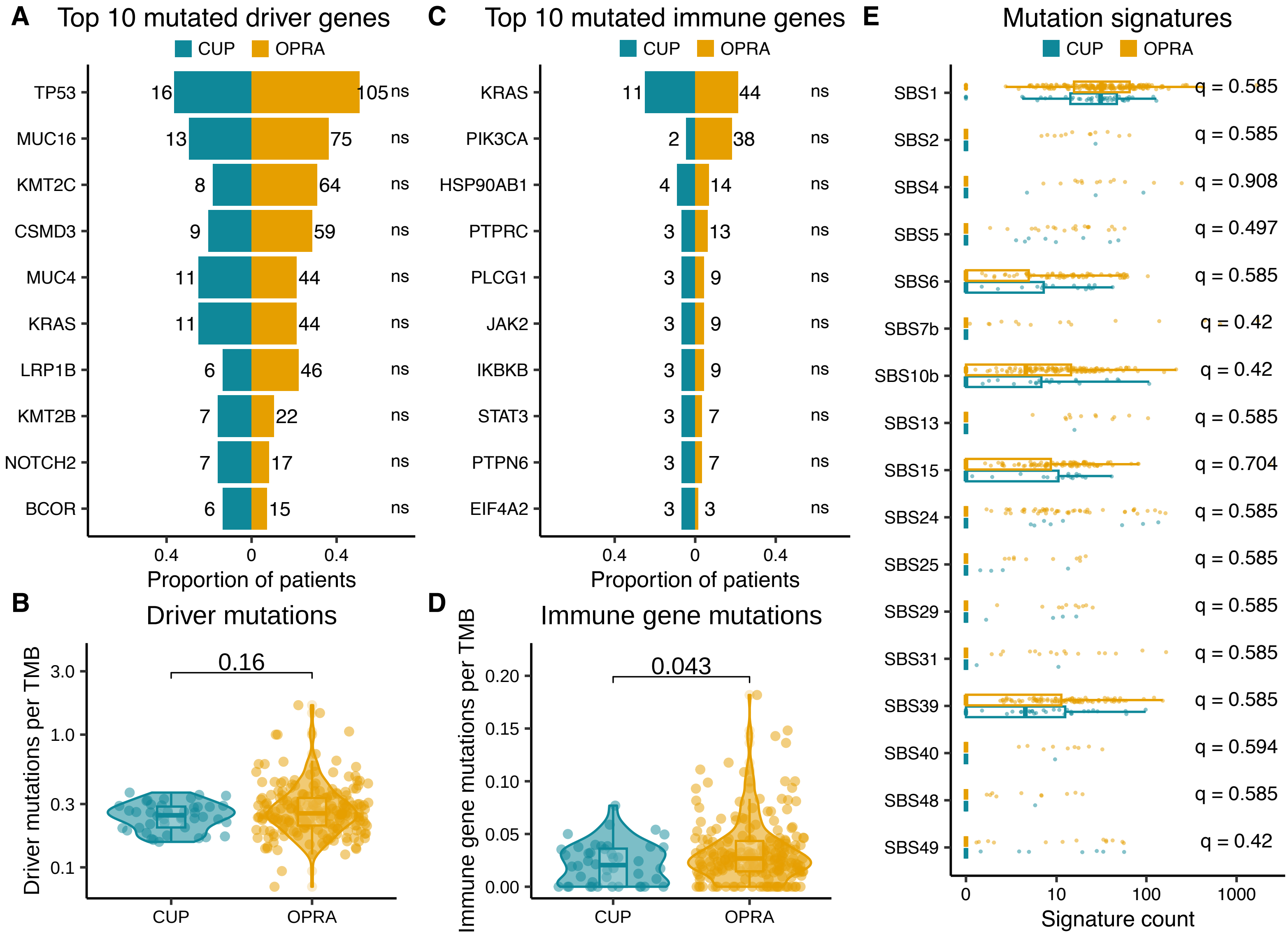

A
Top 10 mutated driver genes
CUP
OPRA
TP53
MUC16
KMT2C
CSMD3
MUC4
KRAS
LRP1B
KMT2B
NOTCH2
BCOR
16
105
13
75
8
64
9
59
11
44
11
44
6
46
7
22
7
17
6
15
ns
0.4
0
0.4
Proportion of patients
C
Top 10 mutated immune genes
KRAS
PIK3CA
HSP90AB1
PTPRC
PLCG1
JAK2
IKBKB
STAT3
PTPN6
EIF4A2
11
44
2
38
4
14
3
13
3
9
3
9
3
9
3
7
3
7
3
3
E
Mutation signatures
SBS1
SBS2
SBS4
SBS5
SBS6
SBS7b
SBS10b
SBS13
SBS15
SBS24
SBS25
SBS29
SBS31
SBS39
SBS40
SBS48
SBS49
q = 0.585
q = 0.585
q = 0.908
q = 0.497
q = 0.585
q = 0.42
q = 0.42
q = 0.585
q = 0.704
q = 0.585
q = 0.585
q = 0.585
q = 0.585
q = 0.585
q = 0.594
q = 0.585
q = 0.42
0
10
100
1000
Signature count
B
Driver mutations
0.16
Driver mutations per TMB
3.0
1.0
0.3
0.1
CUP
OPRA
D
Immune gene mutations
0.043
Immune gene mutations per TMB
0.20
0.15
0.10
0.05
0.00
