# Supplementary material for: Exploring the molecular landscape of cancer of unknown primary: A comparative analysis with other metastatic cancers
Source: Mol Oncol. 2024 May 15;18(10):2393–406. doi: 10.1002/1878-0261.13664 (PMC11459033; doi:10.1002/1878-0261.13664)
Supplement: Supplementary file 1 — Fig. S1. UMAP based on gene expression of all protein coding genes in the cancer of unknown primary (CUP) and Oncology Precision Medicine Aarhus (OPRA) cohorts. Fig. S2. Comparison of exhausted CD8 T cells between cancer of unknown primary (CUP) patients and distinct cancer types of Oncology Precision Medicine Aarhus (OPRA) with 10 or more patients. Fig. S3. Comparison of T cell fractions between cancer of unknown primary (CUP) and distinct cancer types in Oncology Precision Medicine Aarhus (OPRA) with 10 or more patients. Fig. S4. Mutational landscape of the cancer of unknown primary (CUP) (n = 44) and Oncology Precision Medicine Aarhus (OPRA) (n = 207) cohort. Table S1. Overview of biopsy sites for the cancer of unknown primary (CUP) patients (n = 27). Table S2. Differentially expressed genes between the cancer of unknown primary (CUP) (n = 43) cohort and the Oncology Precision Medicine Aarhus (OPRA) (n = 213) cohort. Table S3. Differentially expressed Hallmark gene sets between the cancer of unknown primary (CUP) (n = 43) and Oncology Precision Medicine Aarhus (OPRA) (n = 213) cohort. [file MOL2-18-2393-s001.zip › supplementary_material_legends.docx]

**Supplementary figure legends**

**Figure S1.** UMAP based on gene expression of all protein coding genes in the cancer of unknown primary (CUP) and Oncology Precision Medicine Aarhus (OPRA) cohorts. Colored by distinct cancer types in the OPRA cohort. Shaped by CUP (circle, n=43) and OPRA (triangle, n=213) cohort.

**Figure S2.** Comparison of exhausted CD8 T cells between cancer of unknown primary (CUP) patients and distinct cancer types of Oncology Precision Medicine Aarhus (OPRA) with 10 or more patients. The x-axis shows the given cancer type. The y-axis shows the Danaher exhausted CD8 T cell value (Methods). *: p-value <= 0.05, **: p-value <= 0.01, ***: p-value <= 0.001 (Wilcoxon ranked sum test). Numbers indicate the number of patients in the given cancer type.

**Figure S3.** Comparison of T cell fractions between cancer of unknown primary (CUP) and distinct cancer types in Oncology Precision Medicine Aarhus (OPRA) with 10 or more patients. **A.** Comparison of T cell fraction in blood buffycoat. The x-axis shows the individual cancer types. The y-axis shows the T cell fraction estimated by T cell ExTRECT. **B.** Same as A but in the tumor. *: p-value <= 0.05, **: p-value <= 0.01 (Wilcoxon ranked sum test). Numbers indicate the number of patients in the given cancer type.

**Figure S4.** Mutational landscape of the cancer of unknown primary (CUP) (n=44) and Oncology Precision Medicine Aarhus (OPRA) (n=207) cohort. **A.** Driver mutation analysis of the top 10 mutated driver genes across cohorts. The x-axis shows the fraction of patients with the driver mutation in each cohort. Numbers indicate the number of patients with the given mutation in each cohort. ns = non-significant (Fisher’s exact test). **B.** Comparison of driver mutations. The y-axis shows the number of driver mutations normalized by tumor mutation burden (TMB). Wilcoxon ranked sum test. **C.** Same as A but for immune genes defined as genes listed as being part of the immune system in the Reactome pathway database[[25]](https://paperpile.com/c/l4JgW0/FGvtl). **D.** Comparison of the number of mutations in immune related genes per TMB. Wilcoxon ranked sum test. **E.** Comparison of single base substitution (SBS) signature counts. The x-axis shows the signature count. The y-axis shows signatures where 10 or more patients had a signature estimate > 0. Wilcoxon ranked sum test.

**Table S1.** Overview of biopsy sites for the cancer of unknown primary (CUP) patients (n=27).

**Table S2.** Differentially expressed genes between the cancer of unknown primary (CUP) (n=43) cohort and the Oncology Precision Medicine Aarhus (OPRA) (n=213) cohort. Positive mean transcript per million (TPM) difference indicates increased expression in the CUP cohort. Negative mean TPM difference indicates increased expression in the OPRA cohort. Wilcoxon rank sum test p-values are adjusted for multiple testing using false discovery rate (FDR) adjustment.

**Table S3.** Differentially expressed Hallmark gene sets between the cancer of unknown primary (CUP) (n=43) and Oncology Precision Medicine Aarhus (OPRA) (n=213) cohort. Positive mean gene set variation analysis (GSVA) difference indicates increased expression in the CUP cohort. Negative mean GSVA difference indicates increased expression in the OPRA cohort. Wilcoxon rank sum test p-values are adjusted for multiple testing using false discovery rate (FDR) adjustment.
