# Supplementary figures and images for: Exploring the molecular landscape of cancer of unknown primary: A comparative analysis with other metastatic cancers
Source: Mol Oncol. 2024 May 15;18(10):2393–406. doi: 10.1002/1878-0261.13664 (PMC11459033; doi:10.1002/1878-0261.13664)

Comparing Exhausted CD8 cells between CUP and OPRA cancer types

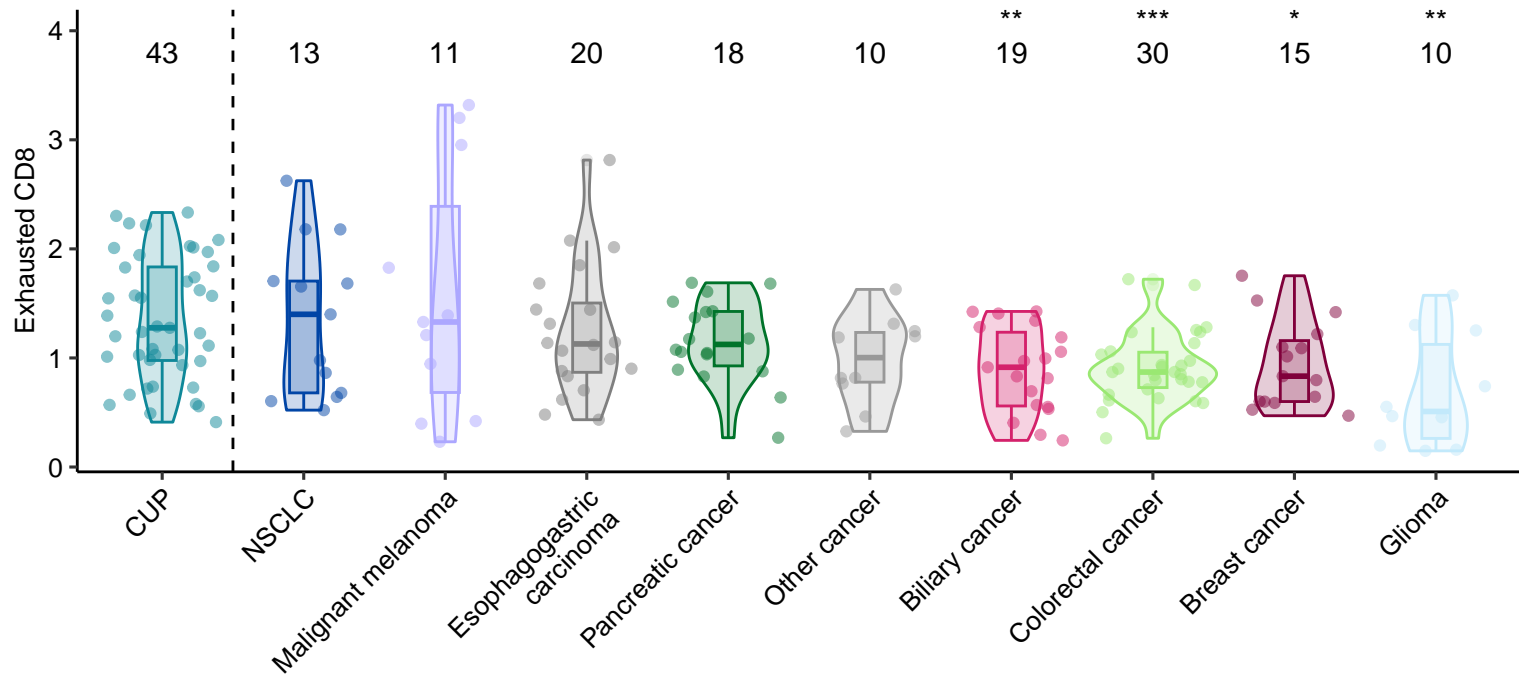

Supplement: Supplementary file 1 — Fig. S1. UMAP based on gene expression of all protein coding genes in the cancer of unknown primary (CUP) and Oncology Precision Medicine Aarhus (OPRA) cohorts. Fig. S2. Comparison of exhausted CD8 T cells between cancer of unknown primary (CUP) patients and distinct cancer types of Oncology Precision Medicine Aarhus (OPRA) with 10 or more patients. Fig. S3. Comparison of T cell fractions between cancer of unknown primary (CUP) and distinct cancer types in Oncology Precision Medicine Aarhus (OPRA) with 10 or more patients. Fig. S4. Mutational landscape of the cancer of unknown primary (CUP) (n = 44) and Oncology Precision Medicine Aarhus (OPRA) (n = 207) cohort. Table S1. Overview of biopsy sites for the cancer of unknown primary (CUP) patients (n = 27). Table S2. Differentially expressed genes between the cancer of unknown primary (CUP) (n = 43) cohort and the Oncology Precision Medicine Aarhus (OPRA) (n = 213) cohort. Table S3. Differentially expressed Hallmark gene sets between the cancer of unknown primary (CUP) (n = 43) and Oncology Precision Medicine Aarhus (OPRA) (n = 213) cohort. [file MOL2-18-2393-s001.zip › mol213664-sup-0002-FigureS2.pdf]

T cells in blood buffy coat

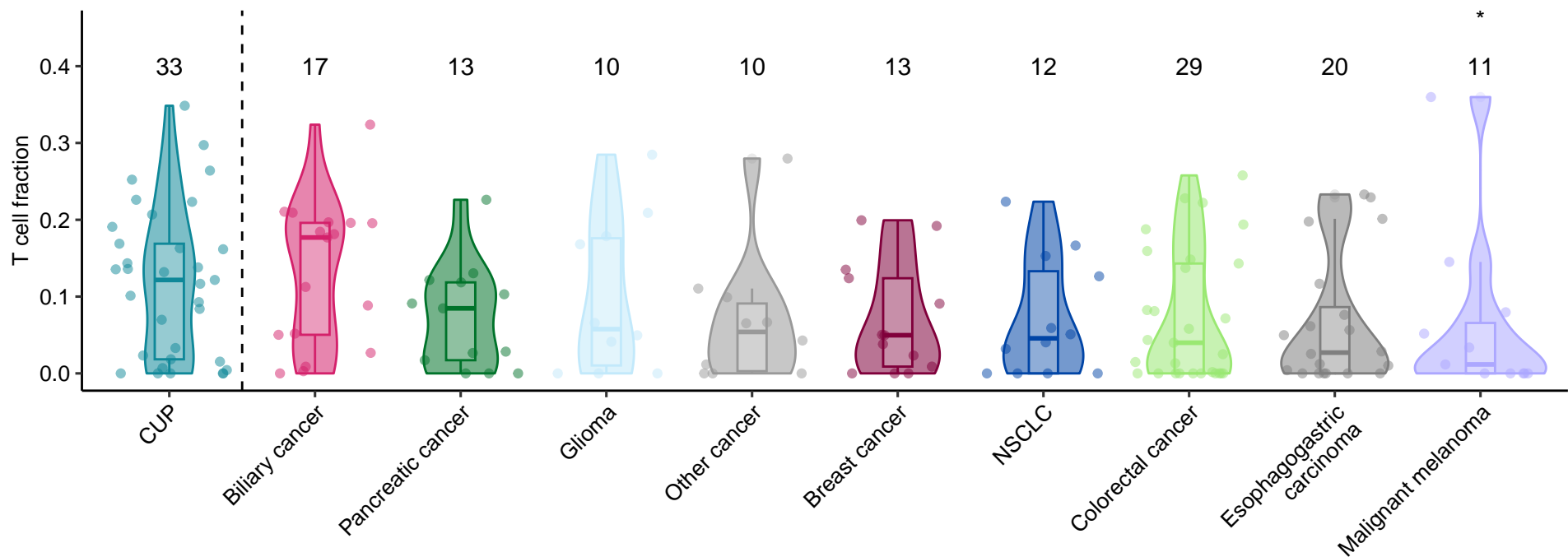

T cells in tumor

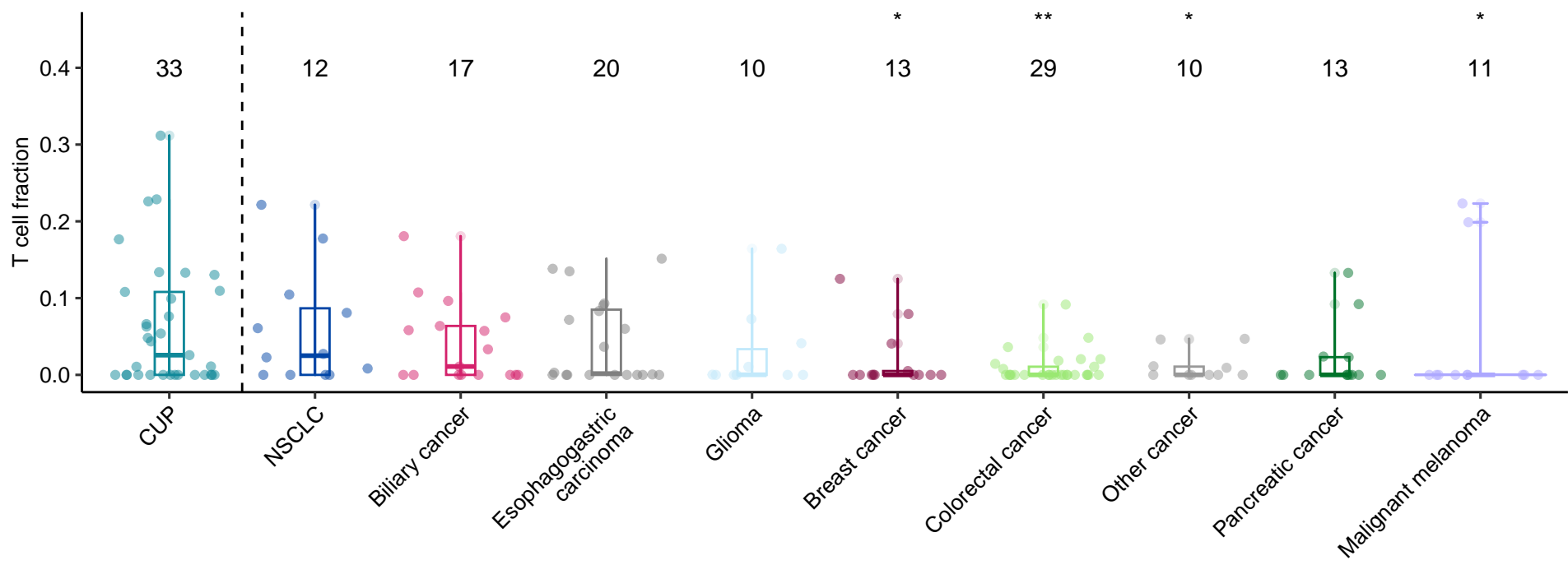

Supplement: Supplementary file 1 — Fig. S1. UMAP based on gene expression of all protein coding genes in the cancer of unknown primary (CUP) and Oncology Precision Medicine Aarhus (OPRA) cohorts. Fig. S2. Comparison of exhausted CD8 T cells between cancer of unknown primary (CUP) patients and distinct cancer types of Oncology Precision Medicine Aarhus (OPRA) with 10 or more patients. Fig. S3. Comparison of T cell fractions between cancer of unknown primary (CUP) and distinct cancer types in Oncology Precision Medicine Aarhus (OPRA) with 10 or more patients. Fig. S4. Mutational landscape of the cancer of unknown primary (CUP) (n = 44) and Oncology Precision Medicine Aarhus (OPRA) (n = 207) cohort. Table S1. Overview of biopsy sites for the cancer of unknown primary (CUP) patients (n = 27). Table S2. Differentially expressed genes between the cancer of unknown primary (CUP) (n = 43) cohort and the Oncology Precision Medicine Aarhus (OPRA) (n = 213) cohort. Table S3. Differentially expressed Hallmark gene sets between the cancer of unknown primary (CUP) (n = 43) and Oncology Precision Medicine Aarhus (OPRA) (n = 213) cohort. [file MOL2-18-2393-s001.zip › mol213664-sup-0003-FigureS3.pdf]
